# Evaluation of physicochemical and phytochemical properties of Safoof-E-Sana, a Unani polyherbal formulation

**DOI:** 10.4103/0974-8490.72332

**Published:** 2010

**Authors:** Ajazuddin, Shailendra Saraf

**Affiliations:** *University Institute of Pharmacy, Pt. Ravishankar Shukla University, Raipur, C.G. - 492 010, India*

**Keywords:** Phytochemical properties, physicochemical properties, Safoof-E-Sana, Tibb-e-Unani, Unani system of medicine

## Abstract

**Background:**

Although the formulations of the Unani system of medicine are popular, not much scientific work has been reported so far. The present article is an attempt to establish the scientific basis of one of the popular Unani formulations Safoof-ESana, a polyherbal formulation widely used as a laxative.

**Methods:**

Investigations were carried out to study the physicochemical and phytochemical properties of Safoof-E-Sana and its active ingredients.

**Results and Conclusion:**

The values of percentage loss on drying, angle of repose, Hausner ratio, and Carr’s index of the formulation were calculated as 8.25 ± 0.582, 27.68, 1.23, and 19 respectively, which indicate that the moisture content of the formulation is within the range and depict good flow characteristics. The total ash, acid- nsoluble ash, and water-soluble ash were found to be 19.146 ± 0.237, 2.351 ± 0.223, and 49.216 ± 0.634, respectively; the value of total ash indicates that the inorganic contents of the formulation are below the limits. Alcoholic and aqueous extracts of the formulation and ingredients were prepared and evaluated for phytochemical analysis and extractive values, and the results show that alkaloids of the formulation are more soluble in water than in alcohol and the higher aqueous extractive value (45.784 ± 0.876) of Unani formulation depicts that water is a better solvent of extraction for the formulation than ethanol.

## INTRODUCTION

In the past decade, there has been renewed attention and interest in the use of traditional medicine (Ayurveda, Naturopathy, Unani, Siddha, and Homeopathy) and yoga globally. It is estimated that 65% of the population in rural India use medicinal plants to help meet primary health care needs.[1] Under the parasol of traditional medicine systems, the Unani system of medicine is also gaining global acceptance due to the amazing clinical efficiency of the formulations. Although Unani medicines have long been used, there is negligible documented evidence regarding their safety and effectiveness. The lack of evaluation has, in turn, slowed down the development of regulations and legislations. Recently, Good Manufacturing Practices (GMP) rules have been proposed for Unani medicines to ensure the quality of the manufactured drugs and gain credibility to make them acceptable globally. With this in view, the department of AYUSH has constituted the Unani Pharmacopoeia Committee for the preparation of the pharmacopoeial standards of single and compound Unani drugs. The Drugs and Cosmetics Act, 1940, controls the standards of manufacturing, sale, and distribution of Unani drugs.[2]

Tibb-e-Unani (Unani medicine) claims to possess many safe and effective drugs, useful in various abdominal disorders. The formulations, despite being widely used in the management of abdominal ailments have not been scientifically studied for their pharmacologic effects and physicochemical evaluation for assurance of uniformity of the quality of formulations. The evaluation of physicochemical properties of the ingredients is essential for the assessment of the quality, that is, ash values determinations, such as total ash, sulfated ash, water-soluble ash, and acid-insoluble ash. The extractive values and phytochemical analysis of the drugs and formulations are also performed to ensure the presence of plant actives and their solubility profile. The polyherbal Unani formulation Safoof-E-Sana and its ingredients have been investigated for the first time in this study for their physicochemical and phytochemical parameters.

Safoof-E-Sana is in a fine powder form (Unani formulation), which is widely used as a laxative at a dose of 3–6 gm/day. It is composed of Burge Sana (Senna leaves), used in constipation, fever, skin diseases, and gout; Zanjabeel (dry ginger) used in asthma, diarrhea, cardiac diseases, and wounds; Poste Haleel e zard (haritakee) used in cardiac diseases, jaundice, cough, and carcinoma; and Namak e Siyah (black salt), which is claimed to posses laxative and carminative properties. The key ingredient of Safoof-E-Sana is senna leaves (*Cassia angustifolia*). All the ingredients are first powdered separately and then mixed together. The genus *Senna* is known to possess important medicinal properties, as it is a rich source of anthraquinones, flavonoids, polysaccharides, sterols, and stilbenoids,[3–6] showing a wide spectrum of biological activity. From the therapeutic point of view, the most important characteristic is the laxative property.[7] The biological activity is related to the anthraquinones and flavonoids in this genus.

In the present study, physicochemical and phytochemical evaluation of the Unani formulation Safoof-E-Sana and its ingredients has been carried out because these evaluations are surprisingly uncharted till date and determination of these parameters are very essential to assure the quality, safety, and efficacy of this formulation.

## MATERIALS AND METHODS

### Materials

All the plant materials, such as senna leaves (*C. angustifolia*), dry ginger (*Zingiber officinale*), haritakee (*Terminalia chebula*), and black salt (Vit lavana or vidam) were purchased from the local market of Raipur, C.G., and identified morphologically and microscopically and compared with standard pharmacopoeia monographs. All the reagents and solvents used were of analytical grade. The ash values, extractive values with various reagents and were determined as per the World Health Organization (WHO) guidelines.[8]

### Preparation of formulation

The plant materials were cleaned by using a sterilized cloth duster to remove dust and by air blowing to remove minute sand particles. The formulation was prepared strictly as prescribed in the book of Unani Pharmacopoeia.[9] Fifty grams of each ingredient, namely, senna leaves, dry ginger, haritakee, and black salt were weighed accurately and made into fine powder by passing through sieve no. 80. The powders were mixed geometrically in a plastic tray and packed in plastic containers.

### Determination of loss on drying

The percentage loss on drying (%LOD) was determined for Safoof-E-Sana and for all the raw ingredients of the formulation because any excess of water in medicinal plant materials will encourage microbial growth, the presence of fungi or insects, and cause deterioration following hydrolysis. The %LOD was determined gravimetrically[8] in which 5 g of accurately weighed air-dried material was placed in a previously dried and tared flat weighing bottle. The sample was dried in an oven at 100°C–105°C until 2 consecutive weighings did not differ by more than 5 mg.

### Bulk density

A sample of about 50 cm^3^ of powder that has previously been passed through a US Standard no. 20 sieve was carefully introduced into a 100 mL graduated cylinder. The cylinder was dropped at 2-s intervals on a hard wooden surface 3 times from a height of 1 in. The bulk density was then obtained [Table 1] by dividing the weight of the sample in g by the final volume in cm^3^ of the sample contained in the cylinder.

**Table 1:** Physicochemical parameters of Safoof-E-Sana and its raw materials

Name	%LOD n= 3	Tap density	Bulk density	Angle of repose	Hausner ratio	Carr’s index
ZO	7.13 ± 0.682	0.57	0.36	34.48	1.58	37
CA	6.34 ± 0.445	0.47	0.30	29.52	1.56	37
TC	7.82 ± 0.474	0.83	0.68	28.92	1.22	18
BS	—	0.42	0.38	22.54	1.10	10
LF	8.25 ± 0.582	0.48	0.39	27.68	1.23	19

ZO, *Zingiber officinale*; CA, *Cassia angustifolia*; TC, *Terminalia chebula*; BS, Black salt; LF, lab formulation; %LOD, percentage loss on drying.

### Tap density

A sample of about 50 cm^3^ of powder that has previously been passed through a U S standard no. 20 sieve was carefully introduced into a 100 mL graduated cylinder.[10] The cylinder was dropped at 2-s intervals on a hard wooden surface hundred times from a height of 1 in until there was no further decrease in the volume of powder. The tap density was then obtained by dividing the weight of the sample in g by the final volume in cm^3^ of the sample [Table 1] contained in the cylinder.

### Angle of repose

A glass funnel was held in place with a clamp on ring support over a glass plate. The glass plate was placed on a micro lab jack. Approximately 100 g of the powder was transferred into the funnel (that has previously been passed through a no. 10 size mesh), keeping the orifice of funnel blocked by the thumb. As the thumb was removed, the lab jack was adjusted so as to lower the plate and maintain about 6.4 mm gap between the bottom of the funnel stem and the top of the powder pile. When the powder was emptied from the funnel, the angle of the heap to the horizontal plane was measured [Table 1] with a protractor.[10] The height of the pile (*h*) and the radius of the base (*r*) were measured using a ruler. The angle of repose was thus estimated by the following formula. Values for angle of repose ≤ 30 usually indicate free flowing material and angle ≥ 40 suggested a poor flowing material.

$$\Phi =\tan^{-1}\left(h/r\right)$$

### Hausner ratio

The Hausner ratio was calculated [Table 1] by the formula given below, where *ρ*B is the freely settled bulk density of the powder and *ρ*T is the tapped density of the powder.[11] Values less than 1.25 indicate good flow and a value greater than 1.25 indicates poor flow.

$$H\;=\;\frac{\mathrm{\rho T}}{\mathrm{\rho B}}\;$$

### Carr index

The Carr index is an indication of the compressibility of a powder. It was calculated [Table 1] by the following formula, where *V*_B_ is the freely settled volume of a given mass of powder, and *V*_T_ is the tapped volume of the same mass of powder.[11] The value below 15% indicates good flow characteristics and a value above 25% indicates poor flow characteristics.

$$C=100\frac{V_{B}-V_{T}}{V_{B}}\;$$

### Qualitative phytochemical studies

To detect the presence of various phytoconstituents in formulation as well as in raw materials, phytochemical investigation was performed. The tests were performed on alcohol and water extracts. Qualitative phytochemical analyses were done for Safoof-E-Sana and all the raw ingredients of formulation.[12] Alkaloids, carbohydrates, glycosides, tannins, and phenolic compounds, flavonoids, fixed oils, saponins, proteins and amino acids, and steroids [Table 2].

**Table 2:** Phytochemical characterization of alcoholic and aqueous extracts of Safoof-ESana and its raw materials

Test	Ethanolic extracts				Aqueous extracts			
	ZO	CA	TC	LF	ZO	CA	TC	LF
Alkaloids	–	–	–	–	–	+	–	+
Carbohydrates	+	+	–	+	+	+	+	+
Glycosides	–	+	+	+	–	–	+	+
Tannins and phenols	+	+	+	+	–	–	+	+
Flavonoids	–	+	–	+	–	+	–	+
Fixed oil	–	–	–	–	–	–	–	–
Saponins	–	–	–	–	–	+	–	+
Proteins and amino acids	–	–	+	+	–	–	+	+
Steroids	–	–	–	–	–	–	–	–

ZO, *Zingiber officinale*; CA, *Cassia angustifolia*; TC, *Terminalia chebula*; LF, lab formulation.

### Determination of ash values

#### Total ash

Four grams of the powdered material was accurately weighed and placed in a previously ignited and tared silica crucible. The material was spread in an even layer and ignited by gradually increasing the heat to a temperature of 500–600°C until it was white, indicating the absence of carbon. The material was cooled in a desiccator and weighed [Table 3]. The content of total ash was calculated in mg/g of air-dried material.

**Table 3:** Percentage ash value of Safoof-E-Sana and its raw materials

Name	Total ash (%w/w) n=3	Acid-insoluble ash (%w/w) n=3	Water-soluble ash (%w/w) n=3
ZO	5.023 ± 0.643	0.567 ± 0.011	12.134 ± 0.883
CA	7.023 ± 0.426	1.023 ± 0.643	34.247 ± 0.648
TC	3.891 ± 0.423	0.423 ± 0.008	50.362 ±0.424
LF	19.146 ± 0.237	2.351 ± 0.223	49.216 ± 0.634

ZO, *Zingiber officinale*; CA, *Cassia angustifolia*; TC, *Terminalia chebula*; LF, lab formulation.

### Acid-insoluble ash

To the crucible containing the total ash, 25 mL of hydrochloric acid was added, covered with a watch glass and boiled gently for 5 min. The watch glass was rinsed with 5 mL of hot water and this liquid was added to the crucible. The insoluble matter was collected on an ashless filterpaper and washed with hot water until the filtrate was neutral. The insoluble matter left on the filter paper was transferred to the original crucible, dried on a hot plate and ignited to constant weight. The residue was allowed to cool in a suitable desiccator for 30 min, and then weighed without delay [Table 3]. The content of acid-insoluble ash was calculated in mg/g of air-dried material.

### Extractive values

The extractive values were recorded in alcohol and water [Table 4] with a view to study the distribution of various constituents of Safoof-E-Sana, and all the raw ingredients of the formulation. Accurately weighed 4.0 g of coarsely powdered air-dried material was placed in a glass-stoppered conical flask and macerated with 100 mL of the solvent for 6 h, shaking frequently, and then allowed to stand for 18 h. The mixture was filtered rapidly taking care not to lose any solvent. Twenty-five milliliters of the filtrate was transferred to a tared flat-bottomed dish and evaporated to dryness on a water bath. The residue was dried at 105°C for 6 h, cooled in a desiccator for 30 min, and weighed without delay.[8]

**Table 4:** Extractive values of Safoof-E-Sana and its raw materials

Name	Alcohol-soluble extractive n=3	Water-soluble extractive n=3
ZO	10.248 ± 0.981	14.232 ± 0.883
CA	5.648 ± 0.228	28.226 ± 3.268
TC	22.21 ± 1.442	49.668 ± 3.102
LF	19.12 ± 1.724	45.784 ± 0.876

ZO, *Zingiber officinale*; CA, *Cassia angustifolia*; TC, *Terminalia chebula*; LF, lab formulation.

## RESULTS AND DISCUSSION

### Physicochemical properties

The moisture content of *Z. officianalis*, *C. angustifolia*, *T. chebula*, and lab formulation was found to be 7.13 ± 0.682, 6.34 ± 0.445, 7.82 ± 0.474, and 8.25 ± 0.582, respectively [Table 1]. The moisture content of the formulation was within acceptable range (5%–8%), thus implying that the formulation can be stored for a long period and would not easily be attacked by microbes. The physical properties, namely tapped density, bulk density, angle of repose, Hausner ratio, and Carr’s index were calculated for Safoof-E-Sana and its raw materials. The values of the angle of repose for raw materials *C. angustifolia*, *T. chebula*, *Z. officianalis*, black salt, and lab formulation were 34.48, 32.57, 29.52, 22.54, and 27.68, respectively, which show good flow properties of the prepared lab formulation. The flow properties are also confirmed by Hausner ratio and Carr’s index [Table 1]. Values of Hausner ratio less than 1.25 indicate good flow (20% Carr’s index) and the value greater than 1.25 indicates poor flow (33% Carr’s index)[13]. Both parameters were determined for prepared Unani formulation and it was found to be 1.23 and 19%, respectively, and indicates good flow characteristics.

### Phytochemical analysis

Results of the phytochemical screening of the raw materials and lab formulation of Safoof-E-Sana are given in Table 2. One notable difference as a result of the methods of extraction is the possibility that the alkaloids in *C. angustifolia* are more water soluble, the reason why the presence of that group was not detectable in the ethanolic extract. Furthermore, where more than one test was conducted for the detection of a chemical group, such as the alkaloids, no differences in the results were observed for the different tests. Out of the 9 phytochemical groups investigated, 5, namely, carbohydrates, glycosides, tannins, and proteins were detected in the ethanolic extract of lab formulation and 7 were present in the aqueous extract of lab formulation, including alkaloids and saponins with the previously stated 5. Fixed oil and steroids were absent in all the ingredients and lab formulation for both methods of extraction.

### Determination of ash value

The total ash values of *Z. officianalis*, *C. angustifolia*, *T. chebula*, and lab formulation were 5.023 ± 0.643, 7.023 ± 0.426, 3.891 ± 0.423, and 19.146 ± 0.237, respectively [Table 3]. The value of total ash in the formulation is comparatively high because of the presence of black salt in the lab formulation. Acid-insoluble ash value of the prepared formulation (2.351 ± 0.223) shows that a very small amount of the inorganic component is insoluble in acid. It indicates that adulteration of raw ingredients by substances, such as silica and rice husk, is very less, and a low acid-insoluble ash value may also affect the amount of the component absorbed in the gastrointestinal canal when taken orally.

### Determination of extractive values

Alcohol-soluble and water-soluble extractive values of ingredients and formulation are depicted in Table 4, which shows 19.12 ± 1.724 alcohol-soluble extractive value and 45.784 ± 0.876 water-soluble extractive value of Unani formulation. Higher water-soluble extractive value implies that water is a better solvent of extraction for the formulation than ethanol.

Although the formulations of the Unani system of medicine are popular, not much scientific work has been reported so far. The present article is an attempt to establish the scientific basis of one of the popular Unani formulations, Safoof-E-Sana. It is a polyherbal Unani formulation widely used as a laxative. An investigation was carried out to study the physicochemical and phytochemical properties of Safoof-E-Sana and its active ingredients. The values of %LOD, angle of repose, Hausner ratio, and Carr’s index of the formulation were calculated as 8.25 ± 0.582, 27.68, 1.23, and 19, respectively, which indicate that the moisture content of the formulation is within the range and depict good flow characteristics. Total ash, acid-insoluble ash, and water-soluble ash were found to be 19.146 ± 0.237, 2.351 ± 0.223, and 49.216 ± 0.634 respectively; the value of total ash indicates that the inorganic contents of the formulation are below the limits. Alcoholic and aqueous extracts of the formulation and ingredients were prepared and evaluated for phytochemical analysis and extractive values. The results show that alkaloids of the formulations are more soluble in water than alcohol and a higher aqueous extractive value (45.784 ± 0.876) of Unani formulation depicts that water is a better solvent of extraction for the formulation than ethanol.

## CONCLUSIONS

WHO has emphasized the need to ensure quality control of Unani formulations by using modern techniques and by applying suitable parameters and standards (WHO, 2007). It is the cardinal responsibility of the regulatory authorities to ensure that the consumers get the medication, with purity, safety, potency, and efficacy. As prescribed by the WHO, evaluations of physicochemical and phytochemical properties are essential to standardize the various Unani formulations. In this connection, the authors investigated the stated parameters of Unani formulation Safoof-E-Sana, which surprisingly is unexplored till date.

This is an attempt to establish the scientific basis of one of the popular polyherbal laxative Unani formulations, Safoof-E-Sana. The physicochemical and phytochemical characteristics obtained confirmed the effectiveness and stability of the formulation and the results obtained show that the medium used for the extraction should be aqueous. These explorations will definitely help to set a standard for this traditional medicine.
